# Daytime-Dependent Effects of Thiamine on the Thiamine Pool and Pyruvate Dehydrogenase Regulation in the Brain and Heart

**DOI:** 10.3390/ijms26178296

**Published:** 2025-08-27

**Authors:** Vasily Aleshin, Nadejda Borisova, Artem Artiukhov, Kurban Tagirov, Olga Solovjeva, Eva Lavrenteva, Nikolay Panin, Maria Maslova, Anastasia Graf

**Affiliations:** 1 Belozersky Institute of Physico-Chemical Biology, Lomonosov Moscow State University, 19991 Moscow, Russia; 2 Department of Biological Chemistry, Sechenov University, 119048 Moscow, Russia; 3 Faculty of Bioengineering and Bioinformatics, Lomonosov Moscow State University, 119991 Moscow, Russia; 4 Faculty of Biology, Lomonosov Moscow State University, 19991 Moscow, Russianastjushka@gmail.com (A.G.)

**Keywords:** diurnal rhythms, pyruvate dehydrogenase, protein phosphorylation, thiamine metabolism, thiamine diphosphate, cerebral metabolism, heart metabolism

## Abstract

Thiamine is a vitamin essential for the function of central metabolic enzymes, of which pyruvate dehydrogenase (PDH) possesses one of the broadest regulations. Diurnal effects of thiamine supplementation on energy metabolism have previously been shown for the rat brain. Here, we report data on the diurnal changes and the effects of thiamine administration to rats on the function of thiamine-dependent enzymes in the cerebral cortex, heart, and liver. The most pronounced diurnal differences were found at the level of cerebral PDH activity. Analysis of PDH phosphorylation in five rat tissues revealed diurnal and thiamine-dependent differences in the cerebral cortex and heart. The expression of tissue-specific PDH kinases PDK3 and PDK4 showed a daytime-dependent response to thiamine administration in the cerebral cortex and heart, respectively. In addition, cardiac PDK4 expression was doubled in the evening, compared to morning. Furthermore, cerebral cortex demonstrated tissue-specific diurnal changes in thiamine diphosphate (ThDP) and monophosphate levels. Elevation of blood, cardiac, and cerebral ThDP was more effective upon the evening thiamine administration. Importantly, only ThDP was elevated in the rat cerebral cortex exclusively after evening thiamine supplementation. Coenzyme role of ThDP and/or other thiamine functions in nerve tissue reduced the existing daytime changes in animal behavior and ECG parameters. The reported data on diurnal regulation of central energy metabolism as well as the diurnal difference in thiamine accumulation in the cerebral cortex, heart, and other tissues are of clinical importance, as high doses of thiamine are used for the treatment of acute thiamine deficiencies and many other mostly neurological diseases in patients.

## 1. Introduction

Thiamine is an essential vitamin (B1), well known for its function as a precursor of coenzyme thiamine diphosphate (ThDP), which is the main component of the thiamine pool [1,2]. ThDP is required for the catabolism of carbohydrates and amino acids and the alpha-oxidation of lipids [1,2,3]. The main ThDP-dependent enzymes are mitochondrial pyruvate dehydrogenase (PDH) and 2-oxoglutarate dehydrogenase (OGDH) multienzyme complexes (PDHC and OGDHC, respectively) and cytoplasmic transketolase (TK). The enzymes are of utmost importance for glucose oxidation, indispensable in the brain, and other energy-requiring organs dependent on mitochondrial function, such as the heart, liver, testes, and kidneys. These tissues thus require substantial amounts of thiamine for their energy metabolism [4,5,6]. In addition to the coenzyme function of ThDP, thiamine and its phosphorylated derivatives can act as allosteric regulators of metabolic enzymes [7,8,9], transcription factors [10], receptors [11,12], and other proteins [13,14,15]. Such non-coenzyme function of thiamine is often underrated, resulting in an underestimation of the thiamine requirements of the organism.

Tissues and organs mentioned above differ in thiamine requirements, resulting in several thiamine deficiency syndromes, such as dry and wet beriberi, Wernicke encephalopathy, and Korsakoff syndrome [16,17]. Most of these diseases are caused by the disrupted function of the central nervous system and the brain, with the heart being the second target. For example, dry beriberi is characterized by peripheral sensory and motor neuropathy, while wet beriberi is linked to heart failure. The risk of thiamin deficiency is increased in patients with renal disease, major surgery, septic shock, and severe burns [16]. Administration of high doses of thiamine is used to treat thiamine deficiency. Additionally, high doses of thiamine showed a beneficial effect for the treatment of Parkinson’s disease [18,19,20], inflammatory bowel disease [21,22], and a few neurological conditions [23,24,25,26].

Importantly, thiamine-dependent metabolism demonstrates diurnal variations, known for other metabolic pathways, particularly glucose metabolism in both the brain and peripheral organs [27,28]. Recently, we have shown the diurnal regulation of thiamine-dependent enzymes [8,29,30]; among these, PDHC not only links glycolysis and the Krebs cycle but also produces acetyl-CoA, a substrate for protein acetylation. The latter, together with phosphorylation and other posttranslational modifications, is known to be extensively involved in circadian rhythms [8,29,30,31,32,33]. Recent findings of circadian oscillations in the expression of PDH kinase 4 (PDK4) in human peripheral blood mononuclear cells and adipose tissue [34] indicates that circadian changes in PDHC activity may involve its phosphorylation, and is tissue-dependent [35], and requires further investigation. Although data on PDHC circadian regulation are scarce, the key role of PDHC in energy metabolism, which is known to oscillate during the day [34,36], makes it a prospective target for directed metabolic regulation. Our current work aims to decipher the molecular mechanisms of the high-dose thiamine regulation of central energy metabolism and its potential diurnal dependence and identify the most responsive tissues in rats, focusing on PDHC function and its regulation by phosphorylation.

## 2. Results

### 2.1. Status of the Blood TK Activity and ThDP Level During the Day, and Their Regulation by Thiamine Administration

Activity of blood TK and its endogenous saturation with ThDP can be used as a good indicator for thiamine deficiency in patients [37]. A proportion of holoenzyme below 80–85% (apoenzyme > 15–20%) is considered an indicator of thiamine deficiency (reviewed in [37,38]).

**Figure 1:**
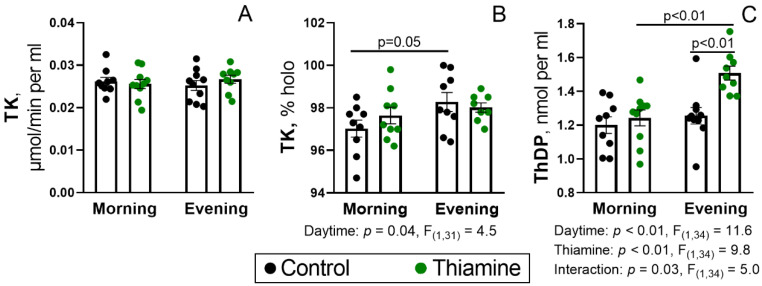
Activity of transketolase (TK) (**A**), its endogenous holoenzyme proportion (**B**), and the level of ThDP in the blood (**C**) and their responses to administration of thiamine in the morning and in the evening. TK activity was measured in the presence of 0.2 mM ThDP; proportion of endogenous TK holoenzyme (% holo) was calculated as the percentage of TK activity without the addition of ThDP to the activity with added ThDP. Each dot corresponds to an individual animal. Thiamine (400 mg/kg) or saline is given to animals in the morning (thiamine—*n* = 10, saline—*n* = 9) or evening (thiamine—*n* = 9, saline—*n* = 10). Data are presented as mean ± SEM. Statistically significant (*p* < 0.05) results of the two-way ANOVA are shown below the graphs as the factors and their interaction with *p*-values and F-statistic values. Differences between the two groups are determined by Tukey’s post-hoc test.

Here, an assay of the blood TK in rats has shown similar values of its total activity independently of the daytime or thiamine supplementation (Figure 1A). No thiamine-deficient rats have been observed, as assessed by the TK saturation. However, the minor daytime effect on the endogenous TK saturation by ThDP indicates it to be higher in the evening (Figure 1B). Moreover, a 25% increase in blood ThDP concentration upon thiamine injection was found only in the evening rats (Figure 1C). Significance of the interaction between the ANOVA factors (*p* = 0.03) indicates a principal difference in the ability of rats to accumulate blood ThDP in the morning and evening after thiamine administration. Thus, these and further data describe the effects of high-dose thiamine on thiamine-sufficient rats, which are clinically relevant due to the thiamine use for the treatment of diseases, not limited to acute thiamine deficiencies [18,22,23,24,25,26,39].

### 2.2. Daytime Dependence of the Thiamine-Dependent Enzyme Activities and Their Tissue-Specific Changes upon the Thiamine Administration

**Figure 2:**
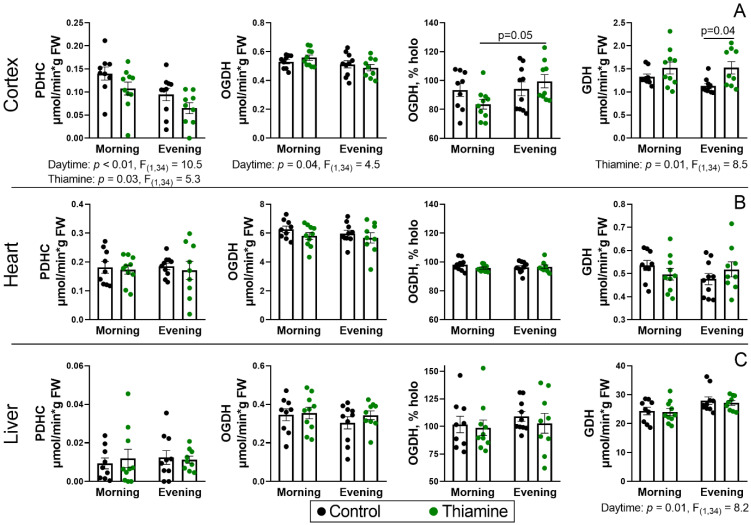
Activities of the ThDP-dependent enzymes and their responses to administration of thiamine in the morning and evening in the rat cerebral cortex (**A**), heart (**B**), and liver (**C**). PDHC—pyruvate dehydrogenase complex, OGDH—2-oxoglutarate dehydrogenase, GDH—glutamate dehydrogenase. Proportion of endogenous OGDH holoenzyme (% holo) was calculated as the percentage of OGDH activity without addition of ThDP to the activity with added ThDP. Thiamine (400 mg/kg) or saline is given to animals in the morning (thiamine—*n* = 10, saline—*n* = 9) or evening (thiamine—*n* = 9, saline—*n* = 10). Data are presented as mean ± SEM. Statistically significant (*p* < 0.05) results of the two-way ANOVA are shown below the graphs as the factors and their interaction with *p*-values and F-statistic values. Differences between the two groups are determined by Tukey’s post-hoc test.

While blood ThDP may represent the average thiamine function of all body tissues, a more precise evaluation of particular tissues would enable a deeper understanding of thiamine regulation in organs with different thiamine demands. Assays of the thiamine-dependent enzymes, including both those regulated by ThDP as a coenzyme and by thiamine or its derivatives as non-coenzyme regulators, were performed in the rat cerebral cortex, heart, and liver. While the brain and heart are the most thiamine-dependent organs due to high energy demands, the liver is the main thiamine storage [5,40,41], in addition to its indispensable metabolic functions. The assessed markers of thiamine function included activities of PDHC, OGDHC, and TK, using ThDP as the coenzyme and GDH and MDH as markers of the non-coenzyme thiamine function [7] (Figure 2 and Figure S1). Among these, only activities of PDHC, OGDHC, and GDH have shown significant differences upon thiamine supplementation or due to the diurnal changes (Figure 2). The differences were observed mostly in the cerebral cortex. Worth noting, PDHC activity corresponds to the maximal flux capacity of the complex, as it is measured with added ThDP and the pyruvate concentration in the assay medium exceeds its endogenous levels [42], while the absence of ThDP in the assay medium results in <10% of the PDHC activity, similar to other studies [43]. Upon the optimal assay conditions, a significantly lower cerebral PDHC activity was detected in the evening (ANOVA factor, *p* < 0.01) (Figure 2A). Thiamine supplementation also resulted in decreased PDHC activity (ANOVA factor, *p* = 0.03). It is worth noting that the observed reduction in PDHC activity does not correspond to PDHC dysfunction observed in fibroblasts of human patients harboring mutations in the genes encoding PDHC components [44]. Such assays are mostly focused on the non-rate-limiting [45,46] decarboxylation of the [1-^14^C]-pyruvate by PDH alone, which, however, is the ThDP-dependent PDHC component, while NADH-dependent pyruvate oxidation was measured in our assay.

The changes in the maximal flux capacity of PDHC were not identical to those detected for OGDHC (Figure 2). While a reduced total OGDHC activity in the evening (ANOVA factor, *p* = 0.04) was similar to PDHC, no changes upon the thiamine supplementation were observed in the former. However, rats receiving thiamine in the morning had 16% lower endogenous OGDHC saturation by ThDP, compared to those receiving thiamine in the evening (Figure 2). The difference likely reflects both a change in total OGDHC activity possessing diurnal changes (ANOVA factor, *p* = 0.04) and a difference in its saturation capacity upon thiamine supplementation. The differences in PDHC and OGDHC activities were accompanied by an increase in cerebral GDH activity upon thiamine supplementation (Figure 2A). The increase was more pronounced (35%) in the evening, although also present (15%) in the morning, resulting in “thiamine” ANOVA factor significance (*p* = 0.01).

The activities of thiamine-dependent enzymes in the heart and liver are similar in all four groups (Figure 2B,C and Figure S1), except for hepatic GDH activity, which is elevated in the evening vs. morning (*p* = 0.01), independent of thiamine supplementation (Figure 2C).

### 2.3. Daytime Changes in the PDH Protein Level and Phosphorylation, and Its Regulation by Thiamine Administration in Rat Tissues

Taking into account the key role of ThDP-dependent PDHC for the rewiring of central metabolism, its potency for regulation, and the revealed changes in its cerebral activity (Figure 2A), we have also screened multiple rat tissues for the daytime- or thiamine-dependent changes in protein expression and phosphorylation of the E1α-subunit of the complex (PDHA protein). Kidney and testicular samples were included in addition to the cerebral cortex, heart, and liver samples to obtain a broader picture of tissue specificity of the results. PDHA Ser293 phosphorylation is considered the most effective regulatory mechanism for the control of PDHC activity. This modification completely inhibits the enzyme [46], providing a reversible mechanism to control the metabolic flux faster than via regulation of protein synthesis.

**Figure 3:**
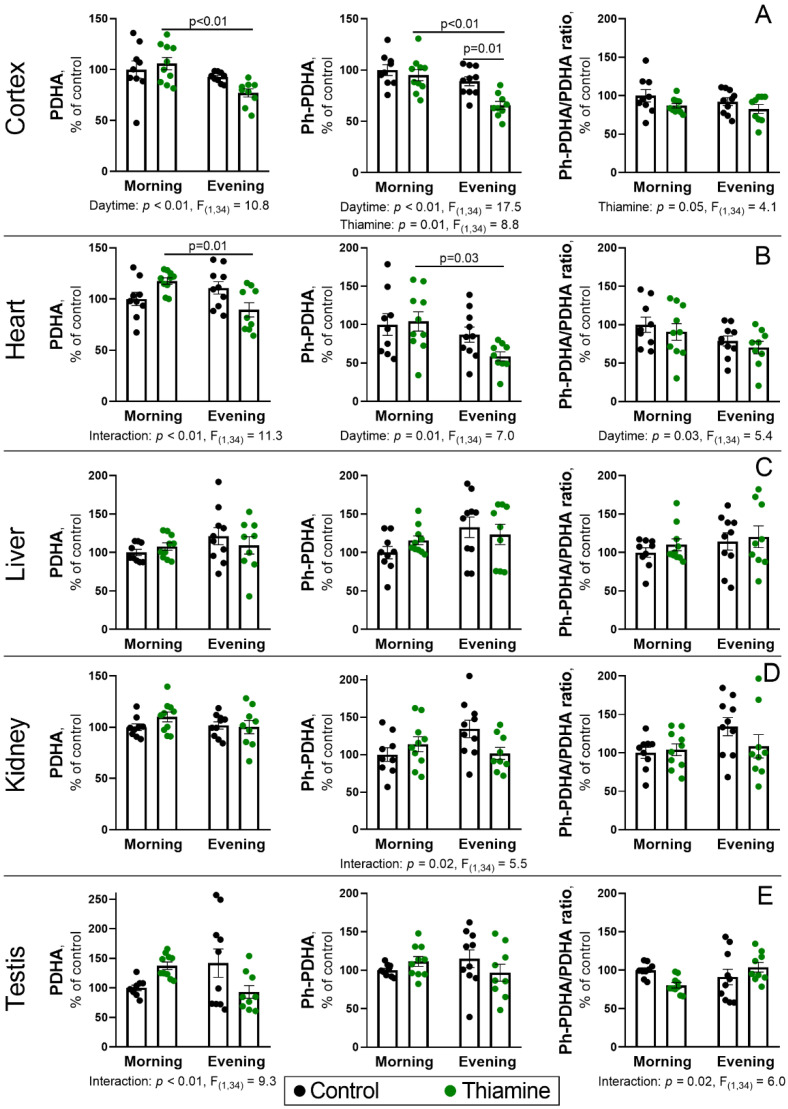
Daytime- and thiamine-dependent regulation of the PDHA level and phosphorylation in rat tissues—cerebral cortex (**A**), heart (**B**), liver (**C**), kidney (**D**), and testes (**E**). PDHA—E1α-subunit of PDHC, Ph-PDHA—phosphorylation of PDHA at Ser293. Ratio between the two parameters (Ph-PDHA/PDHA) was used for the estimation of PDHA phosphorylation level. The parameters were estimated by Western blotting and normalized to the total protein within gel lane, as described in Materials and Methods. Thiamine (400 mg/kg) or saline is given to animals in the morning (thiamine—*n* = 10, saline—*n* = 9) or evening (thiamine—*n* = 9, saline—*n* = 10). Data are presented as mean ± SEM. Statistically significant (*p* < 0.05) results of the two-way ANOVA are shown below the graphs as the factors and their interaction with *p*-values and F-statistic values. Differences between the two groups are determined by Tukey’s post-hoc test.

Similarly to the PDHC activity, cerebral level of PDHA protein is reduced in the evening (*p* < 0.01, ANOVA factor), although the effect is more pronounced for the rats that received thiamine (*p* < 0.01, Figure 3A). Such a pattern largely resembles the one for phosphorylated PDHA (Ph-PDHA). In addition to the reduced level in the evening vs. morning (*p* < 0.01, ANOVA factor), a decrease in Ph-PDHA is observed in thiamine-treated groups, compared to control ones (*p* = 0.01, ANOVA factor). Indeed, the rats that received thiamine in the evening showed only 60% of Ph-PDHA level compared to the control morning group (*p* < 0.01, Figure 3A). In addition, two-way ANOVA has revealed a significant (*p* ≤ 0.05) reduction in the PDHA phosphorylation level calculated as a ratio between Ph-PDHA and PDHA upon thiamine administration (Figure 3A).

Despite the absence of daytime or thiamine effects on PDHC activity in the heart (Figure 2B), its regulation at the protein and phosphorylation levels is substantial and significant in this organ as well as in the cerebral cortex (Figure 3A,B). That is, rats that received thiamine in the evening have lower (*p* = 0.01) PDHA protein level in the heart compared to those that received thiamine in the morning (Figure 3B). The difference between these two groups is also significant (*p* = 0.03) at the levels of Ph-PDHA (Figure 3B) and PDHA phosphorylation (*p* = 0.03, ANOVA factor).

No significant differences in PDHA, Ph-PDHA, or PDHA phosphorylation levels were found in the liver, and only some interactions between ANOVA factors (*p* ≤ 0.02) were observed in the kidneys or testes, with no significance for the factors themselves or significant differences between any of the group means (Figure 3C,D). Nevertheless, all follow similar trends with thiamine administration in the evening, resulting in a minor reduction in the PDHA and Ph-PDHA levels.

Thus, regulation of the daytime changes in PDHA protein level or its Ser293 phosphorylation by thiamine was the most pronounced in the rat cerebral cortex and heart. However, thiamine administration significantly decreases PDHA phosphorylation only in the cerebral cortex while facilitating diurnal changes of cardiac PDHA phosphorylation.

### 2.4. Diurnal Changes and Response to Thiamine Administration of PDH Kinases and Phosphatases and the Components of Thiamine Pool in the Cerebral Cortex and Heart

**Figure 4:**
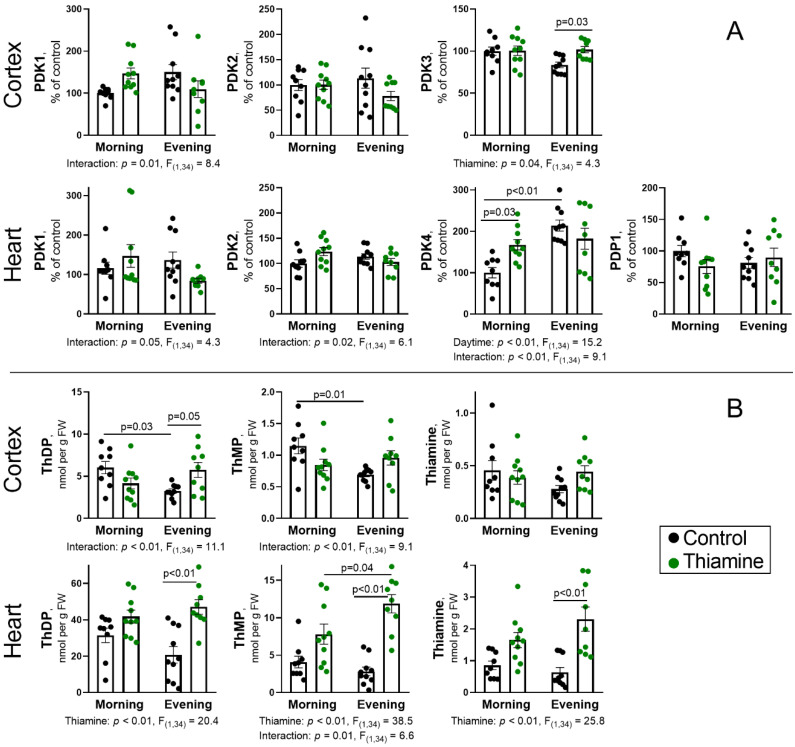
Diurnal changes and response to thiamine administration of (**A**) PDH kinases and phosphatases and (**B**) the main components of thiamine pool in the cerebral cortex and heart. The levels of PDH kinases PDK1, PDK2, PDK3, and PDK4 and PDH phosphatase PDP1 were estimated by Western blotting in the rat cerebral cortex and/or heart, where their levels were detectable. Normalization to the total protein within gel lane is described in Materials and Methods. Thiamine (400 mg/kg) or saline is given to animals in the morning (thiamine—*n* = 10, saline—*n* = 9) or evening (thiamine—*n* = 9, saline—*n* = 10). Data are presented as mean ± SEM. Statistically significant (*p* < 0.05) results of the two-way ANOVA are shown below the graphs as the factors and their interaction with *p*-values and F-statistic values. Differences between the two groups are determined by Tukey’s post-hoc test.

To obtain a broader picture of diurnal PDHC regulation by thiamine in the cerebral cortex and heart, we assessed the levels of regulatory PDH kinases and phosphatases involved in PDHA phosphorylation (Figure 4A), which itself is also regulated by ThDP, inhibiting the PDH kinases. Thus, monitoring of ThDP level (Figure 4B) is inseparable from PDH kinases’ function to understand their regulation of PDHC, while measurement of thiamine and ThMP may enable a deeper understanding of the differences in PDHC regulation in the brain and heart.

Analysis of the PDH kinases and phosphatases with high or intermediate expression in the brain and heart results in the detection of kinases PDK1-3 in the cerebral cortex and kinases PDK1, PDK2, PDK4, and phosphatase PDP1 in the heart. The level of PDK3 is increased by thiamine supplementation (*p* = 0.04, ANOVA factor) in the cerebral cortex (Figure 4A), which is better pronounced in the evening (*p* = 0.03). No significant effects of daytime or thiamine administration were observed on the PDK1 and PDK2 levels in the cerebral cortex, although interaction of the two factors was significant (*p* = 0.01) for the PDK1 (Figure 4A).

The kinases PDK1 and PDK2 revealed no significant effects of daytime or thiamine also in the heart, despite significant interaction of both factors (*p* ≤ 0.05, Figure 4A). PDK4 is highly expressed in the heart but not in the brain of fed animals [35]. This kinase shows a strong daytime effect (*p* < 0.01, ANOVA factor), with twofold higher level in the evening vs. morning; the effect is more pronounced (*p* = 0.03) for the control animals (Figure 4A). Significance of interaction (*p* < 0.01) between the daytime and thiamine factors again indicates a difference between thiamine action during morning and evening injections. Estimation of phosphatase PDP1 expression revealed no significant differences in its level (Figure 4A).

The cerebral levels of major thiamine metabolites, ThDP as well as ThMP, are significantly (*p* ≤ 0.03) reduced in the evening, compared to the morning control rats (Figure 4B). Cortex thiamine level, however, shows no diurnal changes and also remains stable after thiamine supplementation. Instead, thiamine supplementation doubles cerebral ThDP concentration (*p* = 0.05) but only in the evening (Figure 4B), particularly resembling the evening-specific rise of blood ThDP (Figure 1C). The daytime-dependent effect of thiamine supplementation on the cerebral ThDP level and similar pattern for ThMP are manifested in significant interactions between ANOVA factors (*p* < 0.01, Figure 4B).

The changes of thiamine metabolites levels in the heart completely differ from the cortex. All three metabolites are increased after thiamine supplementation (*p* < 0.01, ANOVA factor). The increases in the evening are more pronounced than in the morning for all the three parameters, but ThMP level is significantly higher (*p* = 0.04) in the evening vs. morning thiamine-treated group, also confirmed by significant (*p* = 0.01) interaction between thiamine and daytime ANOVA factors (Figure 4B).

Thus, diurnal changes of thiamine pool without changes in PDH kinases expression are observed in the rat cerebral cortex (Figure 4A,B), which may contribute to the observed diurnal changes in cerebral Ph-PDHA (Figure 3A). Indeed, the daytime-specific effect of thiamine on cortex PDK3 and ThDP levels are observed in the evening, when the change in Ph-PDHA is also significant (Figure 3A and Figure 4).

The revealed diurnal changes in the heart PDH kinases or thiamine metabolites are the most pronounced for PDK4, although its increased level in the evening cannot be responsible for the lower level of Ph-PDHA (Figure 3B and Figure 4). However, the stronger rise in heart ThDP and other thiamine metabolites in the evening vs. morning could contribute to the lower PDHA phosphorylation level in the evening.

Thus, the analyses of thiamine pool and levels of regulatory PDH kinases in the cortex and heart reveal tissue-specific differences between these two main energy-consuming organs. PDK3 and PDK4 were the most affected regulatory PDH kinases of the cerebral cortex and heart, respectively. Cerebral ThDP and also ThMP, but not thiamine or PDH kinases, show diurnal rhythmicity, whereas a strong diurnal shift involves PDK4 level, but not the thiamine pool in the heart. The responses of the cerebral cortex and heart to thiamine supplementation were strongly different as well, especially at the level of thiamine pool metabolites. In general, thiamine injections strongly increased thiamine, ThMP, and ThDP in the heart but not in the cerebral cortex, with the exception of evening ThDP being increased in both tissues. Interaction of diurnal rhythms and different responses to thiamine administration, including higher penetration of ThDP to both organs in the evening, likely influences the PDHA phosphorylation, which, besides the PDHA expression, results in lower Ph-PDHA in the thiamine-supplemented evening vs. morning group (Figure 3A,B).

### 2.5. Daytime Dependence of Physiological Parameters of the Experimental Rats

**Figure 5:**
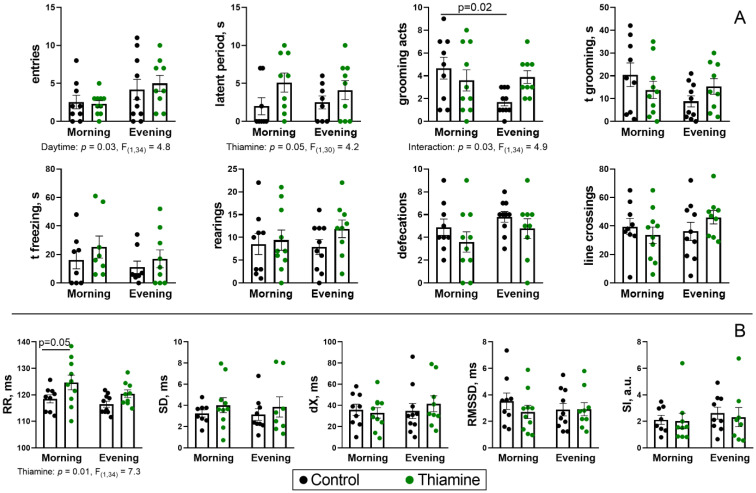
Diurnal assays of the rat behavior (**A**), ECG (**B**), and their responses to the thiamine administration. The ECG parameters (average length of R-R interval and its variability (SD, RMSSD, SI) are as described before [48,49]. Thiamine (400 mg/kg) or saline is given to animals in the morning (thiamine—*n* = 10, saline—*n* = 9) or evening (thiamine—*n* = 9, saline—*n* = 10). Data are presented as mean ± SEM. Statistically significant (*p* < 0.05) results of the two-way ANOVA are shown below the graphs as the factors and their interaction with *p*-values and F-statistic values. Differences between the two groups are determined by Tukey’s post-hoc test.

The potential consequences of the revealed daytime-dependent thiamine action on the key metabolic node PDHC in the rat cerebral cortex and heart are assessed by estimating the previously implemented [47,48] parameters of animal behavior (Figure 5A) and ECG (Figure 5B). Here, diurnal rhythms are observed as a higher (*p* = 0.03, ANOVA factor) number of central entries in the “open field” test (Figure 5A). Such an increase was not affected by thiamine; however, its supplementation increased rat latency by 2–2.5-fold (*p* = 0.05, ANOVA factor) independently of the daytime. Moreover, thiamine supplementation abrogates the daytime difference in the number of grooming acts between control groups (Figure 5A), as indicated by the interaction of the two ANOVA factors (*p* = 0.03). Indeed, evening control rats show half as many grooming acts as morning control rats (*p* = 0.02), but both thiamine-supplemented rat groups show an intermediate number of grooming acts. Other behavioral parameters reveal no significant differences (Figure 5A), whereas among the ECG parameters, only R-R intervals are increased by thiamine injection (*p* = 0.01, ANOVA factor), which is more pronounced in the morning (*p* = 0.05) than in the evening (Figure 5B).

## 3. Discussion

Interpretation of the reported data should take into account that the model animals were thiamine-sufficient (Figure 1B). It accords with the clinical use of thiamine for diseases not limited to those caused by thiamine deficiency, as already mentioned. This partially defined the choice of thiamine dose discussed further. Additionally, while rodents are the first choice for studying thiamine metabolism, their tissues contain 5–10 times higher total thiamine compared to human ones [1,4].

Regarding changes in thiamine metabolism, especially in the cerebral cortex, one should keep in mind the difference between the neuronal and glial compartments. Although our data provide an important piece of evidence for understanding body thiamine homeostasis, the latter is difficult to assess. As a result, the effects observed in tissue homogenates may be specific to some cell populations and more pronounced. In fact, cultured cells do not necessarily represent tissues in situ, and both kinds of data are valuable. Thiamine, ThMP, ThDP, and most enzymes of thiamine metabolism are enriched in neurons vs. glial cells [50], suggesting that most of the processes involving changes in the thiamine pool and function of ThDP-dependent enzymes in the rat cerebral cortex should be attributed to neurons. Such importance of thiamine for neurons is confirmed by the higher sensitivity of viability and acetyl-CoA levels in neurons vs. microglial and astroglial cells to thiamine deficiency [51,52,53], as reviewed in [54]. Higher contribution of thiamine metabolism in neuronal vs. glial cell physiology is especially relevant for the known loss of neurons in Wernicke’s encephalopathy patients and therapeutic potential of thiamine in the treatment of neuropathies related to the loss of neurons, such as Alzheimer’s disease [55].

In addition, of the many assessed metabolic enzymes, including ThDP-dependent TK, PDHC, OGDHC, and the non-coenzyme thiamine function markers GDH and MDH, the study was focused on PDHC. Other enzymes revealed either no (MDH, TK) or small (OGDHC, GDH) effects (Figure 2 and Figure S1), which complement the similar study of metabolism and PDHC function in the cerebral cortex [30]. A 50% reduction in PDHC activity (Figure 2A) and up to a 40% reduction in PDHA protein level in the cerebral cortex (Figure 3A) were in contrast to the almost constant levels of OGDHC activity (Figure 2A). Such a difference in the two ThDP-dependent complexes possessing relatively similar enzyme structures is likely due to the difference in their regulation. Particularly, OGDHC lacks regulation by phosphorylation and is less sensitive to the changes in ThDP level, as it is almost saturated with endogenous ThDP, even upon thiamine deficiency [54,56,57].

Rats are nocturnal animals, which should be taken into account. Our experiment was designed to minimize hyperactivity-driven variability in rats’ behavior, as they exhibit baseline (non-stress-induced) locomotion in the light phase. Still, its variability remains high, and only a few moderate effects of thiamine could be revealed (Figure 5), which, however, are in line with the data on the effects of thiamine administration [8].

Thus, our work provides new data on cerebral metabolic regulation by thiamine, specifically focusing on PDHC as one of the main regulatory targets. The data on the diurnal regulation of the thiamine pool are obtained for the first time. The PDHC regulation and potential connection to the main circadian mechanism are further discussed.

### 3.1. Circadian Regulation of Thiamine, ThDP, and Thiamine-Dependent Proteins

Temporal cycles involving thiamine metabolism are well-known in plants, where thiamine synthesis is controlled by thiamine pyrophosphate (ThDP) riboswitch and the circadian clock [58,59]. The oscillations include not only the level of ThDP, which displays a daily rhythm that is particularly important for the nucleus [59], but also ThMP and thiamine triphosphate levels [58,60]. Thiamine metabolism genes or ThDP level are involved in rhythmic physiological processes such as floret closure as well as stress responses to various stimuli [61,62,63,64].

Less is known about the role of the circadian clock in thiamine metabolism and its regulation in mammals. However, data on the thiamine connection to rhythmic regulation of various cellular processes have been accumulated. Thiamine pyrophosphatase activity, which hydrolyzes ThDP to ThMP and phosphate, exhibits circadian variation in its localization in hepatocytes: the enzyme was detected well in the Golgi apparatus during light but not the dark span. Contrary, the endoplasmic reticulum was positive for the thiamine pyrophosphatase during the dark period, and almost no reaction was observed during the light period [65]. The corresponding enzyme belongs to the apyrase family (human genes *ENTPD1–8*) and has mostly been studied in the brain and liver [2,3,66]. Moreover, altered circadian rhythmicity in murine locomotor activity was shown as an early sign of thiamine deficiency [67]. Pyrithiamine-induced thiamine deficiency disrupted the diurnal rhythm of core body temperature, significantly decreasing it in rats [68]. A similar case of abnormal circadian temperature rhythm was reported in a patient with Wernicke’s encephalopathy [69]. Thiamine supplementation reversed the symptoms and restored the diurnal rhythms in both rodent models and improved the patient’s condition [67,68,69] but also affected the physiological processes in a healthy state. For example, a thiamine analog, sulbutiamin (300 mg/kg), increased the occurrence of fast EEG rhythms in *M. mulatta*, resulting in reorganization of the slow sleep phase and enhanced waking [70]. The corresponding human studies revealed an association of low thiamine intake with excessive daily sleep [71,72], with the association intensified by high alcohol intake and low intake of nutrients, including pyridoxine, niacin, and others. Interestingly, thiamine content in human milk also varied with circadian rhythm, and additional supplementation of thiamine with breakfast resulted in a significant amount of the vitamin passing into milk 2–4 h later [73]. This indicates a circadian rhythm in thiamine (or total thiamine pool) content also in human blood and probably also in urine, as their excretion positively correlates with the blood levels [74].

Our results on the diurnal rhythm of ThDP and ThMP, but not free thiamine, in the rat cerebral cortex, but not in the heart (Figure 4B), provide important evidence for the circadian oscillations in the main thiamine derivatives in mammalian brain.

**Figure 6:**
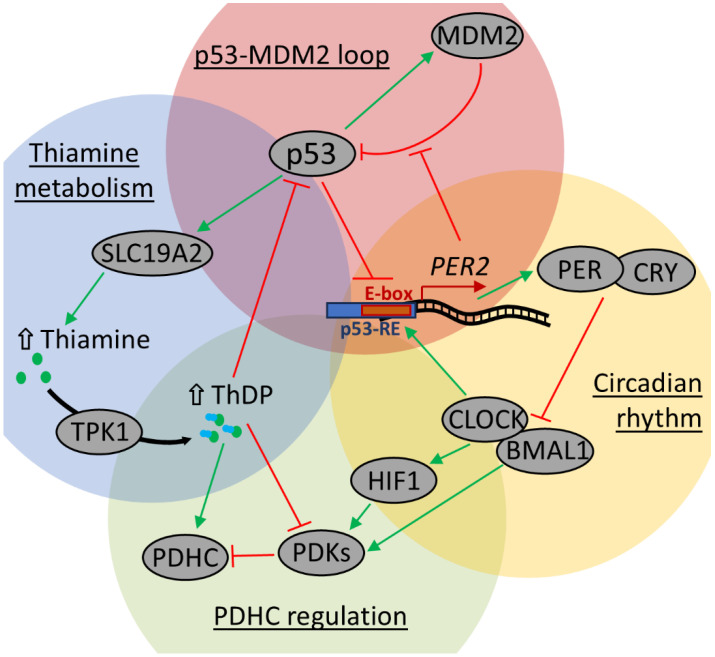
Interconnection between thiamine metabolism and circadian rhythm involves p53 transcription activity. Primary circadian transcription-translation feedback loop involving PER2 (period 2) and CRY (cryptochrome 1 and 2) proteins, as well as E-box enhancer of *PER2* expression, which is activated by heterodimer of CLOCK and BMAL1 proteins [78,82], is shown on a yellow background. A feedback inhibition loop of p53 and MDM2 is shown on a red background: a p53-response element enabling p53-dependent suppression of *PER2* overlaps with the E-box, whereas PER2 suppresses MDM2-dependent ubiquitination and degradation of p53, which activates the MDM2 expression [93]. A part of thiamine metabolism with another feedback inhibition loop is shown on a blue background: p53 induces expression of thiamine transporter SLC19A2, which enables thiamine accumulation, followed by ThDP synthesis via thiamine diphosphokinase (TPK1) [94]. This results in inhibition of p53 transcription activity as well as activation of PDHC directly as its coenzyme or indirectly via inhibition of PDH kinases (PDKs) [9,10]. The latter are also under HIF1-mediated and HIF1-independent transcriptional control by CLOCK and BMAL1 (green background) [78,82,86,88,89,90,92]. Blunt red and sharp green arrows indicate inhibition and activation (or accumulation), respectively.

Such rhythms of ThDP and ThMP in the cerebral cortex but not in the heart (Figure 4B) augment the known oscillations of thiamine-dependent proteins and their regulation, which often involves tissue-specific protein posttranslational modifications [75,76,77]. As we showed previously, this mechanism participates in the diurnal regulation of cerebral GDH [29], pyridoxal kinase [8], and especially PDHC [30,78,79,80,81]. Although the protein levels of the GDH, pyridoxal kinase, and most PDHC components remained constant in the rat cerebral cortex during the day, their modifications display diurnal oscillations. For example, the daily changes in PDHC activity negatively correlated with PDHA Ser293 phosphorylation [30,80]. Most often, this is explained as a result of circadian oscillations either of PDK1 and/or PDK4 expression [78,80,82,83]. The kinases are known to be under transcriptional control by the HIF1α regulator [84,85,86,87], which itself is under control by the main circadian regulators CLOCK and BMAL1 (Figure 6), and its level is known to oscillate during the day [88,89,90]. However, PDK1 and PDK2 promoters were also shown to bind BMAL1 directly [78]. Moreover, the circadian oscillations in both PDK1 and PDK4 and PDHA phosphorylation are disrupted by CLOCK and BMAL1 knockouts and/or functional overexpression [78,80,91]. Finally, PDK1 and PDK4 expression have also been shown to depend on other regulatory circadian proteins (Figure 6), CRY1/2 [78] and PER1 [92].

According to our study, PDK4 seems the most prominent candidate for the circadian regulation of PDHA phosphorylation in the heart, as it has the highest difference between morning and evening rats (Figure 4A). Whereas cerebral PDHC activity changes may be affected more by ThDP availability (Figure 4B) and/or involve other mechanisms, such as calcium oscillations affecting PDP1 activity [79], cyclic changes in the expression of catalytic PDH subunits, encoded by *PDHB*, *DLAT*, or *PDHX* genes [36,82], or phosphorylation of other Ser residues as demonstrated for circannual changes in hibernating ground squirrel during the torpor-arousal cycle [95].

However, the non-coenzyme regulatory thiamine function may be even more susceptible to the changes in ThDP levels than the coenzyme-dependent regulation, because the coenzyme binding is tighter [2]. Among such targets are GDH, pyridoxal kinase, and PDHC. Acetylation of GDH Lys503 changed during the day, which controls the enzyme allosteric regulation by GTP [29,96]. Phosphorylation of pyridoxal kinase Ser213 also showed diurnal changes, which were linked to the levels of protein kinase MAP2K1 and protein phosphatase PPP1CA [8], both linked to circadian regulator gene PER2 [97,98]. Since ThDP is a coenzyme of PDHA, an inhibitor of PDH kinases [9] and pyridoxal kinase [7], and can also regulate the activity of GDH [7], the diurnal oscillation of the cerebral ThDP can participate in direct regulation of these enzymes.

Importantly, daytime oscillations of ThDP level in the brain could participate in periodic protein expression through its direct inhibition of p53 transcription activity [10]. Since p53 itself directly downregulates PER2 through a p53-response element in its promoter [93], higher ThDP could alleviate such p53-dependent inhibition of PER2 expression. The level of p53 oscillates in the mouse suprachiasmatic nucleus, being the highest at ZT 4–8, with PER2 expression being the lowest at 20–4 ZT [93]. Following our data, higher cerebral ThDP in the morning in rats (ZT 2 ± 1; Figure 4B) should correspond to the state of suprachiasmatic nucleus when PER2 expression is low and p53 expression is close to its maximum, while evenings (ZT 9 ± 1) lower ThDP corresponds to nearly the highest PER2 and decreasing amount of p53. In addition to its action on PER2, p53 can induce expression of thiamine transporter 1 encoded by the *SLC19A2* gene [94]. Further conversion of thiamine to ThDP enables feedback inhibition of p53 (Figure 6). Such a mechanism supports the cellular thiamine pool and enables p53-dependent response to thiamine deficiency [99,100,101].

The described connection between the circadian rhythm and oscillation of p53 level also involves the p53–MDM2 feedback loop, which is reciprocally regulated by PER2 [102,103]. Particularly, PER2 binds to p53 and prevents its MDM2-dependent degradation. However, accumulation of p53 downregulates PER2 expression (Figure 6). Thus, the primary circadian transcription-translation feedback loop and ThDP level are connected through transcription activity of the p53 master regulator, and ThDP can both affect PDHC activity and phosphorylation directly and alleviate p53-dependent inhibition of PER2 expression, which again targets PDHC phosphorylation via HIF1-dependent or independent transcription of PDH kinases (Figure 6).

### 3.2. Tissue-Dependent Response of PDHC Regulation and Thiamine Pool to Thiamine Administration

Although the absolute expression of the circadian regulator genes may differ throughout the rat tissues, the oscillation profiles of the key ones, including CLOCK, BMAL1, PER1/2/3, and CRY1/2, are the same in all tissues [82,104]. Thus, the tissue specificity of the daytime dependence of PDHC activity and its changes upon thiamine supplementation is probably dependent on other factors, such as the expression of PDH kinases and oscillation in the thiamine pool.

Diurnal variation of the thiamine metabolites and thiamine-dependent proteins showed differences between tissues. For example, the levels of ThDP and ThMP differed significantly in the morning and evening in the rat cerebral cortex but not in the heart (Figure 4B). The changes in the levels of regulatory tissue-specific kinases (PDK3 and PDK4) upon thiamine supplementation were time-dependent (Figure 4A). PDHC expression, phosphorylation, and activity (Figure 2 and Figure 3) also showed tissue-specific diurnal regulation and response to thiamine supplementation. The tissue dependence of such PDHC regulation is in accordance with the known tissue specificity of PDKs expression, resulting in different dependence of each organ on each PDK. Based on Western blotting [105] and Northern blotting [106,107] analyses, the murine brain, as well as most other tissues, including liver, kidney, and testes, contain mostly PDK2 and, to a lesser extent, PDK3, whereas PDHC function from murine heart and other muscle tissues is dependent on PDK1, PDK2, and somewhat on PDK4. In this regard, PDK4 is exclusive to muscle and adipose tissues, and due to known inducibility by a variety of factors, such as starvation, diabetes, an increase in free fatty acids, and glucocorticoid administration [108], it is a prominent candidate to describe the response of rat cardiac PDHC to thiamine supplementation, whereas PDK3 may mediate the PDHC function in the rat brain.

Our results on the daytime dependence of PDHC regulation may also be complemented by other studies, which seem to show contradictory findings, due to a lack of multiple tissue comparisons within the same experiments, and the strong differences in circadian rhythms even between mice and rats [82]. Nevertheless, the mammalian brain, liver, and kidney seem to have daytime changes in multiple PDHC subunits, including PDK1, PDK3, PDK4, E1β, and E2 [36,78,82], whereas stronger variation in PDHC activity and phosphorylation in skeletal muscle and heart seems to be focused mostly on PDK4 [80,81,82,83,95], which we show here (Figure 4B) and suggested in the previous paragraph. Interestingly, PDK3, whose expression increased with thiamine supplementation and had a significant positive correlation with ThDP content (rS = 0.52, *p* < 0.01), is the major kinase isozyme responsible for inactivation of PDHC in the cerebral cortex. Additionally, negative correlation of PDHC activity and PDK3 level (rS = −0.32; *p* = 0.05) further supports a possible role for PDK3 as a regulatory PDHC kinase in the cerebral cortex, although correlation does not imply causation. The role of PDK4 in muscle tissues of well-fed animals without metabolic disorders is secondary, and the major isozyme in these tissues is PDK2 [105]. Such tissue distribution of PDH kinases is in good agreement with our work, showing positive correlation of PDK2 expression in heart with Ph-PDHA1 level (rS = 0.40, *p* = 0.01) and Ph-PDHA1/PDHA ratio (rS = 0.42, *p* < 0.01).

Among the measured parameters, a striking difference in thiamine ability to enter the cerebral cortex and heart in the morning is especially important due to the metabolic significance of ThDP for the energy supply of these organs. The oscillations in thiamine-dependent proteins are supposed to follow the changes in the cellular thiamine pool, prompting the comparison of thiamine levels and its derivatives between available organs. Although correlation does not imply causation, a correlation analysis showed no association of thiamine, ThMP, and ThDP contents in the heart and cerebral cortex, but the blood ThDP correlated positively (rS = 0.38, *p* = 0.02) with the heart ThDP in the sample of all animals (*n* = 38). Independence of the brain thiamine pool from other tissues may be relevant, taking into account the diurnal variation in cerebral ThDP, multiple sources of diurnal PDHC regulation all affected by ThDP, and its potential link to circadian rhythm (Figure 6). In this regard, the blood–brain barrier, which is permeable for thiamine and ThMP but not ThDP [109], may partially separate the cerebral thiamine pool. Indeed, the significant correlation of the ThDP content in the blood and cerebral cortex (rS = 0.46, *p* = 0.05) only in the thiamine-supplemented groups (*n* = 19) indicate that high level of thiamine can enter into the brain, so ThDP content of the cerebral cortex and erythroid cells become proportional to each other 24 h after administration. However, no correlation was observed between the blood and cerebral ThDP in the control rats. The latter may be related to the diurnal oscillation of cerebral ThDP, not affecting the blood ThDP of control rats (Figure 1C and Figure 4B).

Finally, the brain thiamine transporters of the blood–brain barrier are represented almost solely by SLC19A3, found mostly in brain endothelial cells [110,111,112,113]. Another transporter, SLC19A1, is likely enriched in pericytes [113], although its precise localization in the barrier is not fully clear [111]. While SLC19A3 is a classic thiamine transporter, SLC19A1 mainly acts as a folate transporter that exchanges ThMP or ThDP for folate [2]. Compared to the cerebral cortex, levels of thiamine, ThDP, and ThMP in the heart are more susceptible to external load upon thiamine administration, especially in the evening (Figure 4B). Nevertheless, our data show that accumulation of ThDP in both heart and cerebral cortex, as well as in blood, is greater upon evening administration of thiamine to rats. The existence of such a daytime-dependent difference is important for clinical use.

### 3.3. Potential Clinical Significance of High-Dose Thiamine Supplementation

The dose of thiamine administered to rats in this study (400 mg/kg) is equivalent to the human dose of 64 mg per kg, or about 3.8 g of thiamine per an average weight of 60 kg, according to the formula recommended by the US Food and Drug Administration [29], which suggests dividing the rat dose by 6.2 for the calculation of the human equivalent dose. The dose of 400 mg per kg corresponds to the range of thiamine doses (50–400 mg/kg) used in rodents for the studies of cerebral thiamine levels and their effects [29,114,115]. For humans, however, the calculated dosages are higher compared to the ones used in clinical conditions related to thiamine deficiency, such as Wernicke encephalopathy, Wernicke–Korsakoff syndrome, and others, where the doses normally vary from 100 to 1500 mg (1.7–25 mg per kg) administered for several days [116,117]. Some neurological disorders, however, may require much higher dosages. A month-long therapy with high daily dosages of thiamine in human patients had demonstrated rare side effects of nausea and indigestion at dosages of 7.0–7.5 g/day, respectively [39]. The latter dose corresponds to an approximately 125 mg/kg human dose and an equivalent rat dose of 775 mg/kg, which is nearly double the dose used in this study. Importantly, although excessive thiamine intake is not a major concern, its interaction with other drugs should be considered. Among those is antidiabetic metformin, which is normally taken at dosages of about 0.5 g per day and shares transport routes with thiamine [2], thus potentially affecting saturation of OGDHC and TK with ThDP [56].

Supplementation with high doses of thiamine or its pharmacological derivatives, such as benfotiamine or thiamine disulfide, protects from diabetic dyslipidemia for months, reduces insulin resistance, and prevents obesity in rat experimental models [118,119,120]. In patients, thiamine is most often used for the treatment of Wernicke’s encephalopathy [1,121], however, multiple neurological conditions, including Parkinson’s disease [18,19,20], multiple sclerosis [23], Friedreich ataxia [24], fibromyalgia [25], chronic cluster headache [26], and others (reviewed in [49] and [37]) can also be improved by the administration of high thiamine doses. Finally, thiamine also showed a significant beneficial effect on chronic fatigue in inflammatory bowel disease [21,22] and is used in the therapy of infant patients with genetic disorders of PDHC and branched-chain 2-oxo acid dehydrogenase complex at dosages up to 1200 mg/day [122,123].

A large portion of data on the beneficial effects of high thiamine doses was obtained by Dr. Costantini, who sadly died in 2020. He recommended the early morning and early afternoon dosing of thiamine to avoid any insomnia issues, as his patients often noticed wakefulness after thiamine. Those who received early morning and early afternoon dosing often reported better sleep. These data have been supported by a recent study of thiamine tetrahydrofurfuryl disulfide (TTFD) effects in rats, taking into account that rats are nocturnal animals and humans are diurnal ones. That is, a midday supplementation (ZT 6.5, light phase) of high TTFD dose acutely promoted arousal, reduced sleep, and increased physical activity in rats [124]. The arousal-promoting effect took place not only shortly after the TTFD injection but was also observed on the next morning after the dark phase. A significant association of low thiamine intake with excessive sleep in Korean [71] and Brazilian [72] populations is in line with the arousal promoted by high TTFD [124]. Since rats are nocturnal animals, molecular data showing a better thiamine and/or ThDP accumulation in rat tissues upon evening supplementation (Figure 1C and Figure 4) may suggest a better thiamine/ThDP consumption upon morning supplementation in humans, in accordance with Dr. Costantini’s recommendations and TTFD pharmacological studies. In addition, morning supplementation is also preferred, as it mostly lacks side effects on sleep in humans. Thus, morning supplementation with high-dose thiamine to humans is likely to induce stronger changes in thiamine-dependent metabolism in the brain and heart. However, due to established dependence on circadian rhythms, this corresponds to evening application of thiamine in rodent models.

## 4. Materials and Methods

### 4.1. Materials

Chemicals were obtained from Macklin Biochemical (Shanghai, China) unless otherwise specified. Thiamine hydrochloride was purchased from Sisco Research Laboratories Pvt. Ltd. (Mumbai, India), ThDP (Solarbio, Beijing, China); potassium ferricyanide (purity ≥ 99%) and thiamine monophosphate from Sigma-Aldrich (Steinheim, Germany); and HPLC gradient grade acetonitrile from PanReac AppliChem (Darmstadt, Germany). Throughout all steps of analysis, deionized water purified by an Ultra Clear System (SG Water Conditioning and Regeneration, Barsbüttel, Germany) was used. Buffers and salts were from Helicon (Moscow, Russia). Antibodies used for immunoblotting were from Cell Signaling Technologies (Danvers, MA, USA), CUSABIO (Wuhan, China), FineTest (Wuhan, China), Elabscience (Wuhan, China), and Imtek (Moscow, Russia), as specified in the corresponding section.

A mixture of phosphopentoses for the TK activity and ThDP enzymatic assays was synthesized from ribose 5-phosphate by the established enzymatic procedure using ribose 5-phosphate isomerase and xylulose 5-phosphate epimerase from the rat spleen acetone powder which was shown to have 10–12 units/mL activity of the mixture of ribose phosphate isomerase and xylulose phosphate epimerase, which is identical to the activity of the bovine spleen acetone powder [37,125,126]. Yeast TK apoenzyme was isolated by immunoaffinity chromatography with rabbit polyclonal antibodies and stored in 10 mM potassium phosphate buffer with 50 mM ammonium sulphate at −15 °C according to the published protocol [127,128]. Before the assay, the buffer was changed to 50 mM glycylglycine, pH 7.6, using gel filtration in Sephadex G-50 column (Pharmacia, Uppsala, Sweden), resulting in around 20 U/mg preparation. As conducted previously [37,129], 2.5 mM of CaCl_2_ was added to the TK preparation immediately before the assay to extend the linearity of TK activity on the ThDP concentration [130].

### 4.2. Animal Experiments and Tissue Sample Collection

All animal experiments and tissue collection were carried out according to the Guide for the Care and Use of Laboratory Animals published by the European Union Directives 86/609/EEC and 2010/63/EU and were approved by Bioethics Committee of Lomonosov Moscow State University (protocol number 139-a-2 from 19 May 2022). The study was not pre-registered. Thirty-six Wistar male rats (RRID:RGD_13508588; 13 ± 1 weeks old, 305 ± 15 g) were purchased from the Russian Federation State Research Center Institute of Biomedical Problems of Russian Academy of Sciences (IBMP RAS) and were housed by four or five per cage (555/4K, 580 × 375 × 200 mm), with free access to tap water and standard food for rodents (laboratorkorm.ru) and two-week acclimatization period. The room temperature (22 ± 2 °C), humidity level (53 ± 5%), and a 12 h light/dark cycle (lights from 9:00 a.m. = Zeitgeber time (ZT) 0, to 9:00 p.m. = ZT 12) were under control. Manipulations with the morning groups were performed at 11 ± 1 a.m.; with the evening groups at 6 ± 1 p.m.

**Figure 7:**
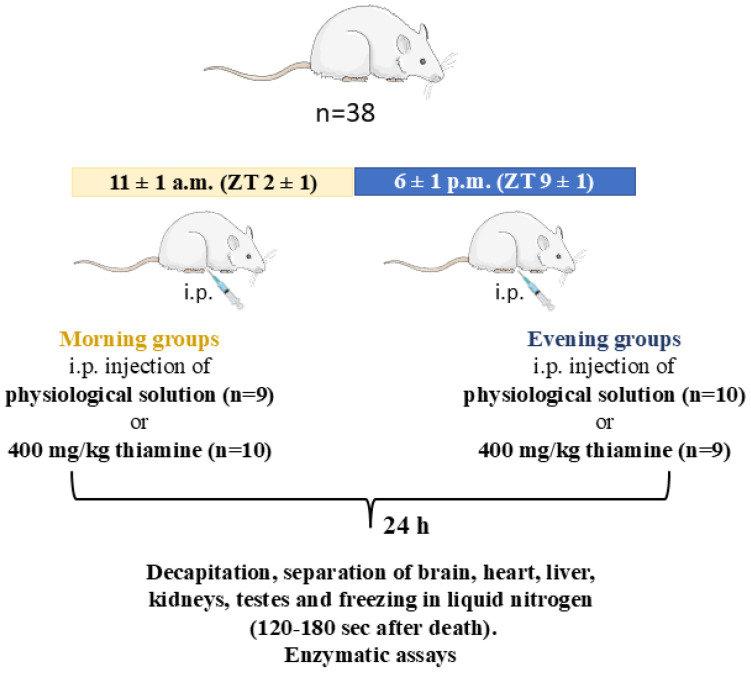
The flowchart of the animal experiment. Wistar male rats (38 rats in total) were randomly divided into four groups. Two of these groups were used for the manipulations in the morning (11 ± 1 a.m., ZT = 2 ± 1), while the other two comprised the evening experimental groups (6 ± 1 p.m., ZT = 9 ± 1). The rats in the treatment groups, either in the morning or evening, were administered thiamine (400 mg/kg) intraperitoneally, whereas the control rats, also divided into the morning and evening groups, were injected with equal volume of saline. The rats were decapitated 24 h after the injections. No animals were excluded from the experiment. The tissue samples, including blood, heart, liver, kidneys, testes, and cerebral cortex, separated from other brain regions, were frozen in liquid nitrogen 60–90 s after the decapitation.

Based on sample size estimation analysis (see “Statistics” chapter), 38 animals were randomly assigned to one of four groups (control morning (CM), thiamine morning (TM), control evening (CE), and thiamine evening (TE)), resulting in nine or ten animals per group (Figure 7). None of the rats died during the experiments or were excluded based on previously used criterion: the rat weight should not differ more than 15% (app. 50 g) from the average on the day before the thiamine or saline injections [29].

The injections were carried out using insulin syringes according to Animal Care Guidelines [131]. The thiamine-treated group received 400 mg/kg intraperitoneal injection of the freshly prepared solution of thiamine hydrochloride in water, pH 6.8–7, as described previously [29]. According to the formula recommended by the US Food and Drug Administration [132], this dose to rats corresponds to 64 mg per kg in humans, which is within the range of doses used in medicine (reviewed in [29]). The control group received a similar injection of saline (0.9% sodium chloride).

Physiological monitoring was performed 24 h after exposure to thiamine or physiological solution. The «Open Field» test («OpenScience», Moscow, Russia) was used to quantify anxiety level, exploratory and locomotor activities, using measures such as grooming, freezing, and latent period durations or number of grooming, defecation, rearing, line crossing, and central entries acts. Electrocardiography (ECG) was registered using non-invasive procedure published [48] to measure the average R-R interval and its variability parameters: parasympathetic, or relaxation, index of the state of the nervous system—RMSSD; sympathetic, or stress, index of the state of the nervous system—SI, required to assess the autonomous regulation of heart rate according to the published method [133,134].

Behavioral assessments (Open Field test and ECG recordings) were conducted 24 h after thiamine/saline administration, during the light phase (morning groups: ZT2 ± 1; evening groups: ZT9 ± 1). Although rats are naturally nocturnal, testing during the light phase was chosen to (i) minimize hyperactivity-driven variability, as rats exhibit baseline (non-stress-induced) locomotion in the light phase; (ii) align with metabolic sampling, ensuring that tissue collection and behavioral tests reflected the same circadian phase.

After physiological monitoring, the animals were decapitated using a guillotine (OpenScience, Moscow, Russia), as described previously [48]. This method of euthanasia was chosen as the most suitable for our studies on the cerebral cortices of adult animals in view of strong interactions of anesthetics with the metabolic changes underlying the state of wakefulness, such as neurotransmitter levels, and with action of neuroprotectants [135,136,137]. The method followed existing recommendations [136] and was approved by Bioethics Committee of Lomonosov Moscow State University. Immediately after the decapitation, the animal brain was excised, and the cerebral cortex separated on ice, followed by freezing in liquid nitrogen 60–90 s after decapitation. The tissue samples were stored at −70 °C. To remove the blood smell and minimize animal stress, the guillotine was washed and cleaned with ethanol after each use.

Blood was collected upon decapitation from the vein into heparin-containing tubes. Aliquots were stored at −70 °C.

Full information on the groups was not available to each of the experimenters involved. For instance, one researcher filled syringes with thiamine or saline, while another one performed the injections substances, not knowing what was in the syringe. Then, 24 h after the injections, one researcher tested the animals in the Open Field, and another one recorded the ECG. The tissue homogenization and extraction procedures, as well as biochemical assays, were also performed without knowing group affiliations of the animal samples.

### 4.3. Preparation of the Rat Tissue Homogenates

Frozen tissues were homogenized and solubilized according to the previously published protocols [29,49]. Briefly, approx. half tissue sample was homogenized using Ultra-Turrax T10 basic (IKA, Staufen, Germany) in the cold homogenization buffer (50 mM MOPS buffer, pH 7.0, containing 2.7 mM EDTA 20% glycerol, and the cocktail of protease inhibitors: 1 mM AEBSF, 0.8 mM aprotinin, 50 mM bestatin, 10 mM pepstatin A, 15 mM E-64, and 20 mM leupeptin) (Solarbio, Beijing, China). A 100 mL aliquot of the homogenate was sonicated using a Bioruptor (Diagenode, Liege, Belgium) on ice-cold water bath, followed by adding one third volume of solubilization buffer (40 mM Tris-HCl buffer, pH 7.4, including 600 mM NaCl, 4 mM EDTA, 1% sodium deoxycholate, and 4% NP-40 (Solarbio, Beijing, China). The mixture was incubated on ice for at least 20 min before assays. Blood samples were also sonicated before the assays, but were not mixed with the solubilization buffer.

### 4.4. Assays of Enzyme Activities in Tissue Homogenates

The enzyme activities of OGDHC and PDHC were measured using CLARIOstarPlus multimodal plate reader (BMG LABTECH, Ortenberg, Germany) in fluorometric mode (340/470 nm), TK using the same reader in spectrophotometric mode (340 nm), and glutamate (GDH) and malate (MDH) dehydrogenases using Sunrise spectrophotometric (340 nm) plate reader (Tecan, Vienna, Austria).

OGDHC was assayed in the rat tissues as described in [48,138,139], in medium containing 50 mM MOPS, pH 7.0, 1 mM dithiothreitol, 1 mM MgCl_2_, 1 mM CaCl_2_, 50 µM coenzyme A, 2.5 mM NAD^+^, 2 mM 2-oxoglutarate, and none or 1 mM ThDP. The reaction was initiated by adding 2.5 μL homogenate (heart homogenate with the highest OGDHC abundance [140] was diluted 5 times in 50 mM MOPS, pH 7.0). PDHC was assayed in similar medium [30,47], except oxamate was used to inhibit both lactate dehydrogenase [141,142] and transaminase [143] reactions, consuming pyruvate, which allows the determination of product (NADH) accumulation rate directly, without coupled reactions with tetrazolium dyes. The reaction was also initiated by adding 2.5 μL of homogenate (diluted 5 times for the heart) into the medium containing 50 mM KH_2_PO_4_, pH 7.5, 3.2 mM L-carnitine, 1 mM dithiothreitol, 1 mM MgCl_2_, 50 µM coenzyme A (Solarbio, Beijing, China), 2.5 mM NAD^+^, 5 mM pyruvate, 25 mM oxamate, and none or 0.2 mM ThDP.

The blood TK activity was measured in a coupled reaction as described previously [37,129]. The sonicated blood samples were diluted 5 times with the assay buffer (50 mM glycylglycine, pH 7.6, 2.5 mM MgCl_2_), and preincubated with the assay buffer also containing 1 mM NADH, 13.5 U/mL triosephosphate isomerase, 0.9 U/mL glycerol-3-phosphate dehydrogenase, and none or 0.2 mM ThDP for 20–40 min in a glass tube until the rate of background decrease in the NADH absorbance becomes constant. This was followed by mixing 50 µL of the blood in the described pre-reaction medium with 150 µL of the assay buffer, also containing 4 mg/mL phosphopentose mixture, and a 90 min assay.

GDH and MDH activities were measured in assay mixtures containing (100 mM Tris-HCl, pH 7.5, 2.5 mM 2-oxoglutarate, 0.2 mM NADH, and 50 mM NH_4_Cl) and (100 mM Tris-HCl, pH 7.5, 0.3 mM oxaloacetate, and 50 mM NH_4_Cl), respectively [138]. Both reactions were also initiated by the addition of homogenates (3 μL for GDH, except liver, which was diluted 5 times; 2 μL for MDH, but all homogenates were diluted 150 times).

Reaction rates in media omitting pyruvate (PDHC reaction), 2-oxoglutarate (OGDHC and GDH reactions), oxaloacetate (MDH reaction), or phosphopentose mixture (TK reaction) were used as blanks and subtracted during reaction rate calculations. Activities were expressed as μmol of product formed per min per g of tissue fresh weight using calibration curve for NADH, linear at 0.01–0.1 nmol/well interval for the fluorometric assays or its molar extinction coefficient of 6220 M^−1^ × cm^−1^ for the spectrophotometric ones. All enzyme activities were measured at 25 °C.

### 4.5. Western Blotting

Cerebral cortex homogenates were diluted in Laemmli buffer and subjected to SDS-PAGE. The resulting gels were used for the assessment of total protein expression via 2,2,2-tricholoroethanol staining, after which the proteins were transferred to PVDF membranes, followed by their 1.5 h incubations with primary and secondary antibodies and chemiluminescent detection as described before [139]. The blocking solution was TBST containing 5% BSA, with BSA concentration decreased to 0.5% for antibody dilution buffer. The following antibodies were used: rabbit anti-PDHA1 (CST (Danvers, MA, USA) #3205, 1:2000), rabbit anti-pSer293-PDHA1 (CST #37115, 1:2000), rabbit anti-PDK1 (FineTest (Wuhan, China) #FNab06275, 1:2000), rabbit anti-PDK2 (CUSABIO (Houston, TX, USA) #CSB-PA003732, 1:2000), rabbit anti-PDK3 (CUSABIO #CSB-PA613585ESR2HU, 1:2000), rabbit anti-PDK4 (Elabscience (Houston, TX, USA) #E-AB-53270, 1:2000), rabbit anti-PDP1 (CUSABIO #CSB-PA833247, 1:1000), and HRP-linked goat anti-rabbit IgG (Imtek (Moscow, Russia) #P-GAR Iss, 1:5000). Raw images of protein bands in membranes and gels are presented in Supplementary Figure S2.

Chemiluminescence and fluorescence signals were detected using ChemiDoc MP Imager (Bio-Rad, Hercules, CA, USA) and processed in Image Lab software v. 6.0.1 (Bio-Rad, Hercules, CA, USA). The band intensities (peak areas) were normalized to total protein in the corresponding gel lane. When samples of one group were processed across several membranes, the normalized intensities were averaged based on the staining of several samples repeated across all the membranes.

### 4.6. Quantifications of Thiamine, Thiamine Monophosphate (ThMP), and ThDP in Tissue Extracts

Blood ThDP was extracted by heating procedure and measured enzymatically using yeast TK apoenzyme as described in [37,144,145]. Briefly, the sonicated blood was diluted 5 times with the TK assay buffer, incubated at 95 °C for 3 min, and centrifuged at 21,500× *g* for 15 min. The supernatant (40 µL) was incubated with 10 µL of the assay buffer containing 3 µg of yeast apo-TK for 40 min followed by addition of 150 µL of the same assay buffer containing 0.33 mM NADH, 4.5 U/mL triosephosphate isomerase, 0.3 U/mL glycerol-3-phosphate dehydrogenase, and 4 mg/mL mixture of potassium salts of xylulose 5-phosphate and ribose 5-phosphate, and measurement of TK reaction rate in the resulting mixture for 30–40 min. The calibration curve was linear when using 40 µL of 0–0.2 µM ThDP solution (0–8 pmol ThDP per microplate well), with actual ThDP concentration in the standard solution determined using ThDP molar extinction of 7500 M^−1^ × cm^−1^ at 272 nm [146].

Methanol-acetate extraction of metabolites from the rat cerebral cortex was performed as described before [147]. Extraction of metabolites from the heart samples was done identically. Briefly, frozen tissue samples were homogenized in 8 vol. of ice-cold methanol, followed by addition of 1.5 vol. of 0.2% acetic acid solution to methanol homogenate and protein precipitation by centrifugation. Thiamine, ThMP, and ThDP quantification in methanol-acetic extracts of rat cortices was performed using modified HPLC with pre-column oxidation of thiamine and its derivatives to thiochrome or the corresponding derivatives and their fluorometric detection (370/435 nm), similar to previous approach [148]. Thiochrome and its derivatives were separated using Kromasil Eternity 5-C18 4.6 × 250 mm column (E05CLA25, Kromasil, Bohus, Sweden) with Series 200 UV/Vis HPLC System (Perkin Elmer, Shelton, CT, USA), Series 200 Peltier Column Oven (Perkin Elmer, Shelton, CT, USA) set at 30 °C, and Fluor-305 fluorescence detector (PerSeptive Biosystems, Framingham, MA, USA). The column was protected by a corresponding guard column of the same material (E05CLNGC, Kromasil, Bohus, Sweden) and equilibrated for 30 min with mobile phase before the injection of samples. The mobile phase consisted of a 5 mM potassium phosphate buffer, pH 6.8, with 9% acetonitrile. Derivatization was performed automatically by adding 11 µL of oxidating reagent (15% NaOH and 30 mM potassium ferricyanide) to 100 µL of methanol-acetate extract, stored on ice, and put into autosampler within 1 min prior to derivatization. After 4 min mixing, 20 µL of sample was injected, followed by elution for 22 min at 1 mL/min flow rate.

Standard solutions of thiamine (50–500 nM), ThMP (2–500 nM), and ThDP (0.2–2 µM) were prepared freshly by dissolving in 0.1 M HCl, and calibration curves were made each day before and after the assays of samples. The calibration curves appeared to be linear within the used range. Peaks were integrated by the software Multichrom v. 3.4 (Ampersand Ltd., Moscow, Russia) [149].

### 4.7. Statistics

Statistical analysis was performed using Prism, version 8.0 (GraphPad Software Inc., La Jolla, CA, USA) or STATISTICA 10.0 (StatSoft GmbH, Hamburg, Germany). The necessary sample size calculation was based on our previous experience with the effects of thiamine administration on enzyme activities in the cerebral cortex [29]. A two-tailed t-test using a power of 80% and a level of significance of 0.05 estimated the groups to require eight animals at minimum.

Based on the results of the D’Agostino and Pearson’s omnibus normality test, comparisons between multiple experimental groups (CM vs. TM vs. CE vs. TE) were made using two-way ANOVA with Tukey’s post hoc test. The ROUT test for outliers did not exclude any data points. Spearman’s correlations were used for the correlation analysis in order not to be confined to linear relationships.

## 5. Conclusions

Our data point to a daytime-dependent difference in the accumulation of thiamine pool components and metabolic changes 24 h after thiamine administration to rats, as well as tissue-specific diurnal changes in ThDP and ThMP levels in the cerebral cortex of control rats. The most pronounced metabolic differences are observed in the protein levels, activity, and phosphorylation of PDH, especially in the cerebral cortex and heart. Further studies are required to better characterize the mechanisms of the diurnal regulation of thiamine transport and/or conversion to ThDP and the proposed role of the primary circadian transcription-translation feedback loop. An extensive analysis of the PDH kinases and phosphatases suggests PDK3 and PDK4 to be involved in the regulation of PDH complex in the cerebral cortex and heart, respectively, enabling a more specific characterization of their roles upon circadian rhythms. We believe our data would enable a better understanding of the metabolic and physiological changes upon the administration of high thiamine doses to patients who are not limited to Wernicke’s encephalopathy and other disorders caused by thiamine deficiency.

## Data Availability

The data presented in this study are available in this article and the Supplementary Materials.

## Supplementary Materials

The supporting information can be downloaded at: https://www.mdpi.com/article/10.3390/ijms26178296/s1.

## Abbreviations

The following abbreviations are used in this manuscript:

ECG	electrocardiography
GDH	glutamate dehydrogenase
MDH	malate dehydrogenase
OGDH	2-oxoglutarate dehydrogenase or α-ketoglutarate dehydrogenase
OGDHC	2-oxoglutarate dehydrogenase complex or α-ketoglutarate dehydrogenase complex
TK	transketolase
PDH	pyruvate dehydrogenase
PDHC	pyruvate dehydrogenase complex
ThDP	thiamine diphosphate, or thiamine pyrophosphate, or cocarboxylase
ThMP	thiamine monophosphate
TTFD	tetrahydrofurfuryl disulfide
ZT	Zeitgeber time
