# Supplementary material for: Daytime-Dependent Effects of Thiamine on the Thiamine Pool and Pyruvate Dehydrogenase Regulation in the Brain and Heart
Source: Int J Mol Sci. 2025 Aug 27;26(17):8296. doi: 10.3390/ijms26178296 (PMC12428021; doi:10.3390/ijms26178296)
Supplement: Supplementary file 1 [file ijms-26-08296-s001.zip › ijms-3796995-supplementary.pdf]

Daytime-dependent effects of thiamine on the thiamine pool and pyruvate dehydrogenase regulation in the brain and heart

Vasily Aleshin, Nadejda Borisova, Artem Artiukhov, Kurban Tagirov, Olga Solovjeva, Eva Lavrentieva, Nikolay Panin, Maria Maslova, and Anastasia Graf

Supplementyary figures

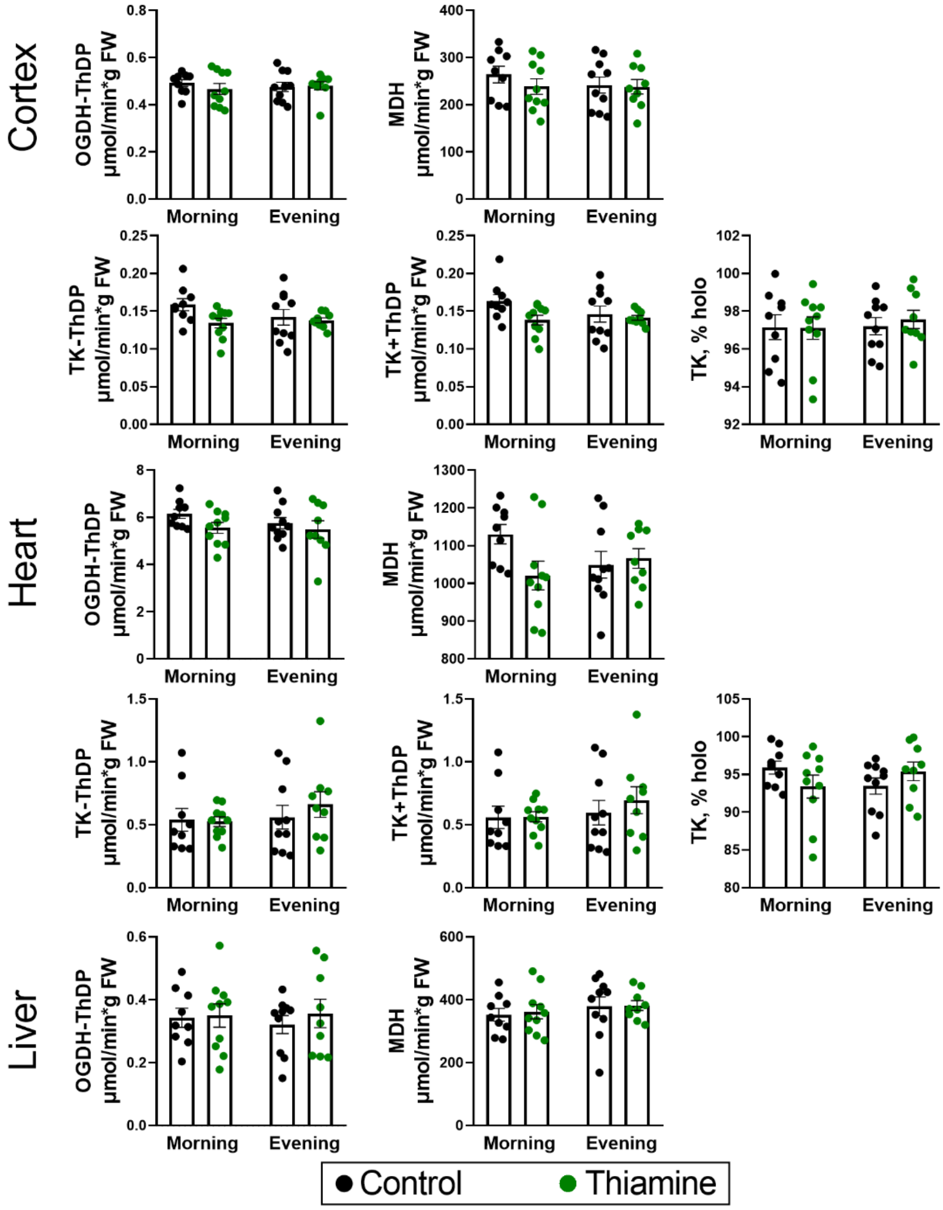

Figure S1. Supplementary data on activities of the ThDP-dependent enzymes and their responses to administration of thiamine in the morning and evening in the rat cerebral cortex, heart and liver. OGDH – 2-oxoglutarate dehydrogenase, MDH – malate dehydrogenase, TK - transketolase. Proportion of endogenous TK holoenzyme (% holo) was calculated as the percentage of TK activity without the addition of ThDP to the activity with the added ThDP. Thiamine (400 mg/kg) or saline are given to animals in the morning (thiamine – n=10, saline – n=9) or evening (thiamine – n=9, saline – n=10). Data are presented as mean ± SEM. Statistical significance was tested using two-way ANOVA and Tukey's post-hoc test, showing no significant ($p<0.05$) results for these enzyme activities.

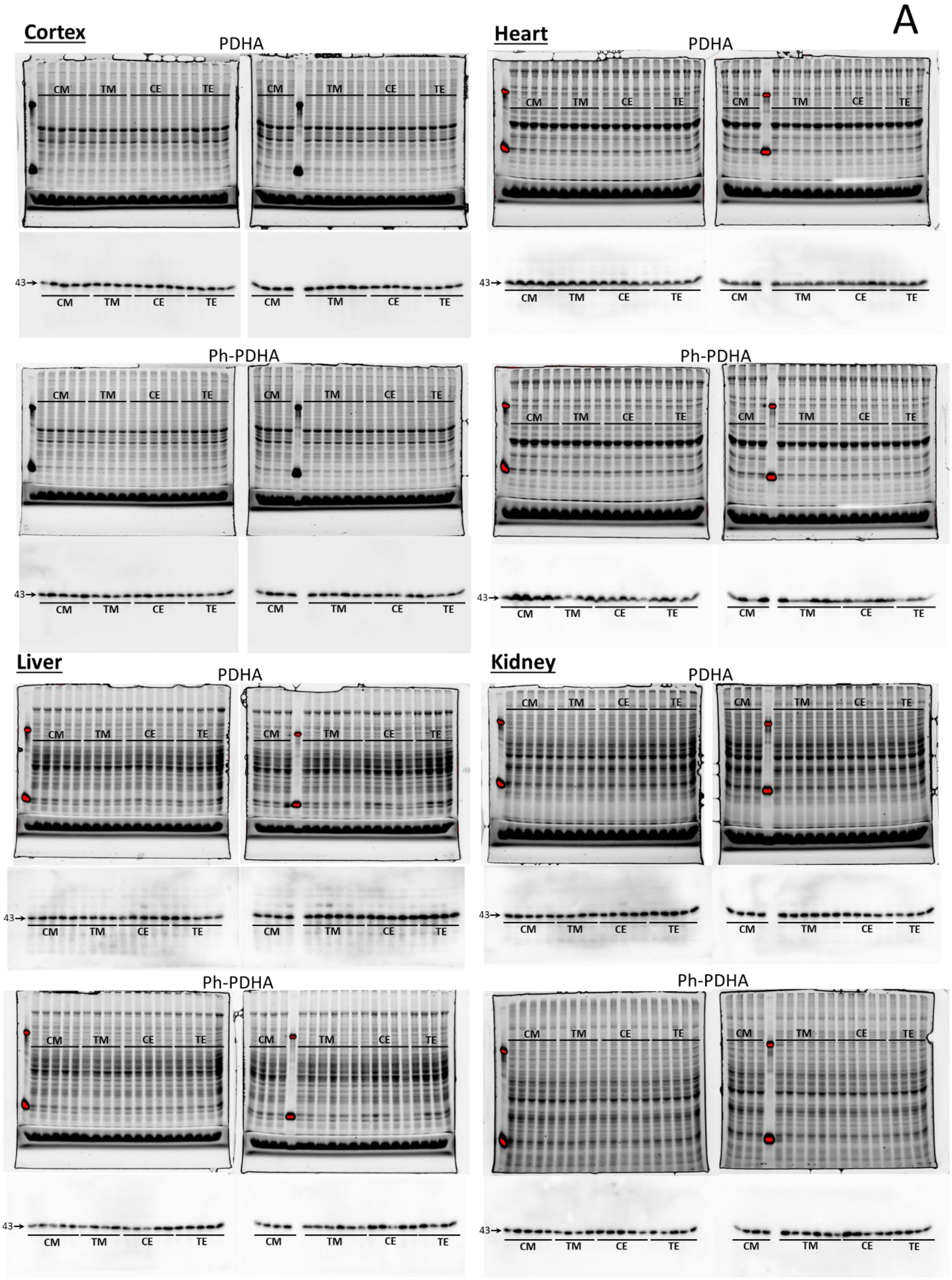
A
Cortex
PDHA
Ph-PDHA
Heart
PDHA
Ph-PDHA
Liver
PDHA
Ph-PDHA
Kidney
PDHA
Ph-PDHA
CM
TM
CE
TE
43→

Testis

PDHA

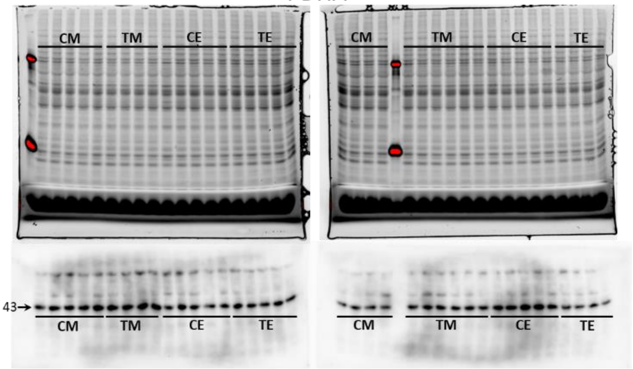

Ph-PDHA

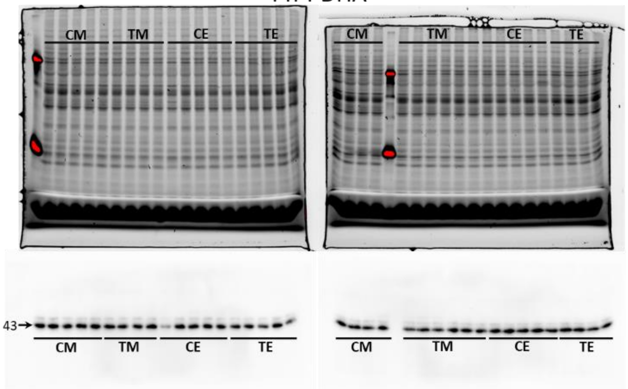

Cortex

B

PDK1

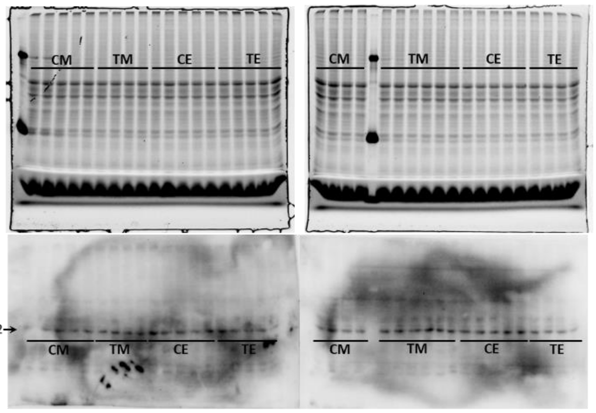

PDK2

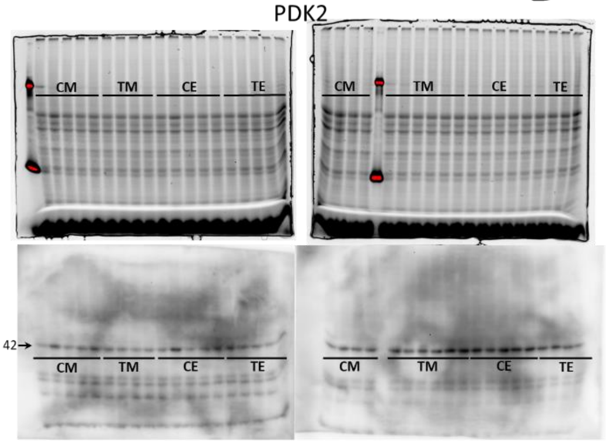

PDK1 repeat

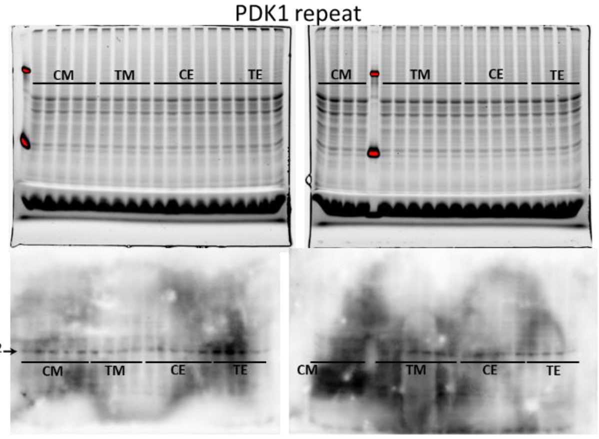

PDK3

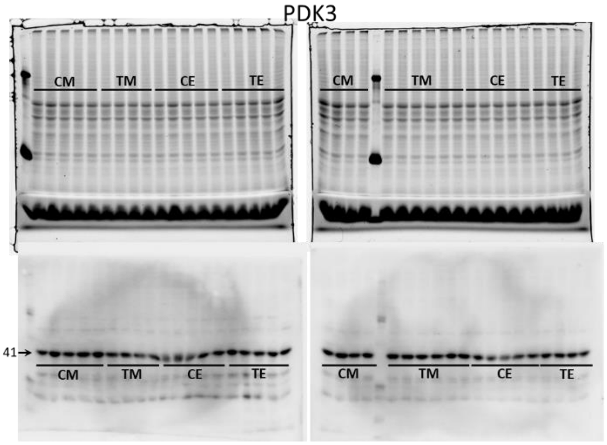

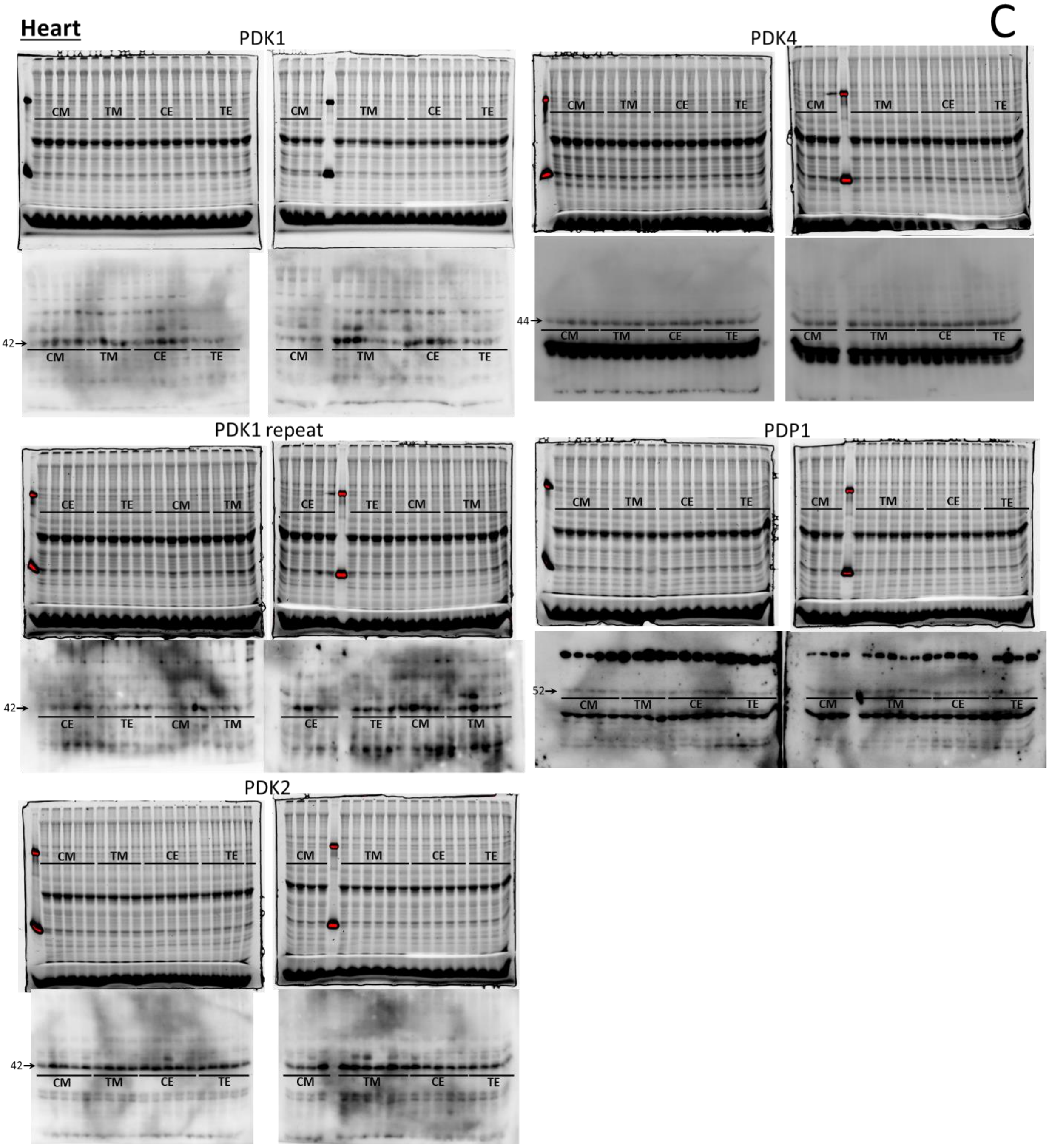

Figure S2. Full-size images of membranes and corresponding gels. (A) – PDHA and Ph-PDHA chemiluminescent detection in rat tissues; (B) – PDH kinases and a phosphatase in rat cerebral cortices; (C) – PDH kinases and a phosphatase in rat hearts. PDHA – E1α-subunit of PDHC, Ph-PDHA – phosphorylation of PDHA at Ser293, PDK1, PDK2, PDK3 and PDK4 – PDH kinases, PDP1 – PDH phosphatase 1. The bands corresponding to the evaluated protein are indicated with arrows and their MW. Thiamine (400 mg/kg) or saline are given to animals in the morning (thiamine – n=10 (TM), saline – n=9 (CM)) or evening (thiamine – n=9 (TE), saline – n=10 (CE)).
